# Retraction statement: A kinase‐interacting protein 1 may serve as a potential biomarker for deteriorative tumor features and poor prognosis in gastric cancer patients

**DOI:** 10.1002/jcla.23683

**Published:** 2021-01-02

Retraction: A kinase‐interacting protein 1 may serve as a potential biomarker for deteriorative tumor features and poor prognosis in gastric cancer patients by Rongbo Lin, Shen Zhao, Liyu Su, Xiaohui Chen, Chunwei Xu, Qinliang He, Changhua Zhuo and Yunbin Ye, *J Clin Lab Anal*. 2020, 34:e23350 (https://onlinelibrary.wiley.com/doi/10.1002/jcla.23350).

The above article, published online on July 16, 2020, in Wiley Online Library has been retracted by agreement between the authors, the journal's Editor‐in‐Chief, Junming Guo, and Wiley Periodicals, LLC. The retraction has been agreed because upon re‐evaluation of the AKIP1 expression data, the authors found that it was originally misread and thus that the conclusions of the study are not valid.

